# 

**Published:** 1866-09

**Authors:** 


### The Chicago Medical Journal.

E. L. HOLMES, M. D., H. M. LYMAN, M.D., R. M. LACKEY, M.D. . . . . Editors.

All letters for the JOURNAL should be addressed to R. M. LACKEY, M.D., P. O Box 2175, Chicago, Ill. Office—169 South Dearborn Street.

TERMS—$2 PER ANNUM, IN ADVANCE.

$3 if not paid before 1st of July of each year. Single Copies, 25 cents.

